# Risk of cancer, cardiovascular disease, thromboembolism, and mortality in patients with rheumatoid arthritis receiving Janus kinase inhibitors: a real-world retrospective observational study using Korean health insurance data

**DOI:** 10.4178/epih.e2023045

**Published:** 2023-04-15

**Authors:** Hong Ki Min, Hyeongsu Kim, Ho Jin Jeong, Se Hee Kim, Hae-Rim Kim, Sang-Heon Lee, KunSei Lee, Soon-Ae Shin, Jong Heon Park

**Affiliations:** 1 Division of Rheumatology, Department of Internal Medicine, Konkuk University Medical Center, Seoul, Korea; 2 Department of Preventive Medicine, Konkuk University School of Medicine, Seoul, Korea; 3 Research Institute of Medical Science, Konkuk University School of Medicine, Seoul, Korea; 4 Big Data Strategy Department, National Health Insurance Service, Wonju, Korea

**Keywords:** Rheumatoid arthritis, Cardiovascular disease, Venous thromboembolism, Cancer, Janus kinase inhibitor, Tumor necrosis factor inhibitor

## Abstract

**OBJECTIVES:**

This study investigated whether Janus kinase inhibitors (JAKis) raise the risk of cardiovascular disease (CVD), venous thromboembolism (VTE), and cancer in patients with rheumatoid arthritis (RA).

**METHODS:**

We conducted a real-world retrospective observational study using data obtained from the Korean National Health Insurance Service database. Two data sets were analyzed: tumor necrosis factor inhibitor (TNFi)/JAKi-naive RA patients (set 1) and all RA patients who used TNFis or JAKis (set 2). The incidence rate ratios (IRRs) and hazard ratios (HRs) for acute myocardial infarction (AMI), stroke, cardiovascular (CV)-related mortality, major adverse cardiovascular events (MACE), VTE, arterial thromboembolism (ATE), cancer, and all-cause mortality were compared between the JAKi and TNFi groups.

**RESULTS:**

Set 1 included 1,596 RA patients (JAKi group: 645; TNFi group: 951), and set 2 included 11,765 RA patients (JAKi group: 2,498; TNFi group: 9,267). No adverse events (AEs) showed significantly higher IRRs in the JAKi groups than in the TNFi groups of sets 1 and 2. The HRs for MACE in the JAKi groups of sets 1 and 2 were 0.59 (95% confidence [CI], 0.35 to 0.99) and 0.80 (95% CI, 0.67 to 0.97), respectively. The JAKi group of set 2 showed a significantly higher risk of all-cause mortality (HR, 1.71; 95% CI, 1.32 to 2.20), but the other AEs did not demonstrate increased risks in the JAKi groups.

**CONCLUSIONS:**

In this study, JAKis did not increase the risk of AMI, stroke, CV-related mortality, MACE, VTE, ATE, or cancer in Korean RA patients relative to TNFis.

## GRAPHICAL ABSTRACT

[Fig f1-epih-45-e2023045]

## INTRODUCTION

Synovitis and progressive destruction of the small joints are the hallmarks of rheumatoid arthritis (RA), a type of autoimmune arthritis [1]. The incidence rates of comorbidities and mortality are higher in RA patients than in the general population [2,3], and cardiovascular disease (CVD), respiratory diseases, and cancers are the leading causes of mortality in this group of individuals [2,4]. In addition, the risk of venous thromboembolism (VTE) is 1.5-2.0 times greater for RA patients than for the general population [5,6]. A chronic inflammatory status contributes to the initiation and progression of atherosclerosis, in addition to inducing a prothrombotic state in inflammatory arthritis [7].

Various medications, including non-steroidal anti-inflammatory drugs (NSAIDs), glucocorticoids, and disease-modifying antirheumatic drugs (DMARDs), are used to treat RA patients [8]. For RA patients who do not respond to conventional synthetic (cs) DMARDs, the American College of Rheumatology, European Alliance of Associations for Rheumatology, and Korean College of Rheumatology recommend initiating therapy with biologic (b) DMARDs or Janus kinase inhibitors (JAKis) [9-11]. The recent Oral Rheumatoid Arthritis Trial (ORAL) Surveillance study of RA patients showed that tofacitinib (a JAKi) was associated with a greater risk of cancers and major adverse cardiovascular events (MACE) than tumor necrosis factor inhibitors (TNFis) [12]. However, the ORAL Surveillance study included older individuals (aged 50 years or over) with at least 1 CVD risk factor, and most of the patients were Caucasian [12]. Two studies analyzing insurance claims data from the United States and France showed that JAKi use did not increase the risk of MACE or VTE in real-world data [13,14]. Furthermore, the risk of MACE was increased only in RA patients with baseline atherosclerotic cardiovascular disease (ASCVD) and not in RA patients without ASCVD, according to a post hoc analysis of the ORAL Surveillance study [15]. Based on the results of the ORAL Surveillance study, the U. S. Food and Drug Administration (FDA), European Medicines Agency, and Korean FDA limited the use of all JAKis to RA patients for whom JAKi treatment was the only option.

In the present study, we aimed to measure and compare the incidence and hazard ratios (HRs) of acute myocardial infarction (AMI), stroke, cardiovascular (CV)-related mortality, MACE, VTE and arterial thromboembolism (ATE), cancer, and all-cause mortality associated with JAKi versus TNFi use in patients with RA, using Korean nationwide health insurance data.

## MATERIALS AND METHODS

### Data sources

Korean National Health Insurance Service (NHIS) data were used in this study. The NHIS is a government-led medical insurance system that covers 97.2% of the Korean population. Reimbursement claims data for healthcare services are collected and recorded in the National Health Information Database (NHID). The NHID has collected health screening, socioeconomic and demographic variables, and mortality data from the entire population of Korea since 2002 [16]. Claims data include information relating to the diagnosis and the associated procedures and prescriptions, which are identified using the International Classification of Diseases, 10th revision (ICD-10) codes and Korean Drug and Anatomical Therapeutic Chemical codes. Data on RA patients who were treated with JAKis or TNFis, based on information in their completed health insurance claims forms, were extracted from the NHIS database (approval No. NHIS-2022-1-648).

### Study population

The present study was a retrospective observational study. Patients with RA were defined as individuals who had: (1) seropositive RA (ICD-10 code: M05) or seronegative RA (ICD-10 codes: M060, M062, M063, M068, or M069) code as their first or second diagnosis code at least twice in either inpatient or outpatient visits; and (2) a prescription of methotrexate (MTX) for at least 6 months after being initially coded as RA [13,17]. Two data sets were collected: (1) patients with newly diagnosed RA who had not yet received bDMARD or JAKi treatment (set 1); and (2) all patients with RA who used JAKis or TNFis after the first JAKi, tofacitinib, was approved in Korea (set 2). Tofacitinib was approved for use in RA patients on March 1, 2015, for patients who did not respond to one or more TNFis. After July 1, 2017, tofacitinib could be given to TNFi-naive RA patients who did not achieve a clinical response to MTX after at least 6 months of treatment. To identify newly diagnosed RA patients who were not exposed to either bDMARDs or JAKis (set 1), patients were selected who: (1) had an ICD-10 code for seropositive or seronegative RA as their first or second diagnosis between January 1, 2014, and December 31, 2020; and (2) used MTX for at least 6 months before initiation of JAKi (i.e., tofacitinib, baricitinib, or upadacitinib) or TNFi (i.e., etanercept, adalimumab, infliximab, or golimumab) treatment. In exclusion step 1, RA patients were excluded from set 1 who had an ICD-10 code for seropositive RA or seronegative RA between January 1, 2014, and December 31, 2016, to select bDMARD-native and JAKi-naive RA patients who were prescribed TNFi or JAKi after July 1, 2017. In exclusion step 2, subjects were excluded who had a diagnosis code for ankylosing spondylitis, systemic lupus erythematosus, psoriasis, psoriatic arthritis, Behçet’s disease, Crohn’s disease, or ulcerative colitis as their first or second diagnosis at least twice. In exclusion step 3, patients with a main ICD-10 diagnosis code for AMI, stroke, VTE, ATE, or cancer 12 months prior to the initiation of TNFi or JAKi were excluded. Patients below 18 years of age were excluded in exclusion step 4. In exclusion step 5, RA patients who did not use JAKis or TNFis, or who started JAKis/TNFis before July 1, 2017, were excluded. The inclusion and exclusion criteria for set 2 were identical to those of set 1; however, for set 2, the first exclusion step (patients diagnosed with RA between 2014 and 2016) was not performed. In addition, in the last exclusion step for set 2, RA patients who started JAKis/TNFis before March 1, 2015, were excluded. Because information on previous bDMARD exposure history was not offered by the Korean NHID, set 2 included both bDMARD-naive and bDMARD-exposed RA patients. The ICD-10 codes used for the exclusion criteria are listed in Supplementary Material 1, and flowcharts outlining the processes of inclusion and exclusion for sets 1 and 2 are presented in Supplementary Materials 2 and 3. Information about comorbidities, including hypertension (HTN), diabetes mellitus (DM), chronic kidney disease (CKD), and heart failure, was obtained from the healthcare utilization database of the NHIS at the start of follow-up. All subjects were followed until December 31, 2021, or until each primary adverse event (AE; i.e., AMI, stroke, CV-related mortality, MACE, VTE, ATE, cancer, or allcause mortality) occurred. The follow-up duration was separately calculated for each primary AE. The last follow-up date was determined as follows: (1) if an event occurred, the date when the event first occurred; or (2) if event-free, until December 31, 2021, or the last date of JAKi/TNFi use; or (3) if switching from JAKi to TNFi or TNFi to JAKi, until the date of the switch. In the case of switching from one TNFi to another TNFi or from one JAKi to another JAKi, these patients remained in the TNFi or JAKi group. However, if switching to another bDMARD, the date of the switch was set as the end of follow-up.

### Definition for adverse events (acute myocardial infarction, stroke, cardiovascular-related mortality, major adverse cardiovascular event, venous thromboembolism, arterial thromboembolism, cancer, and all-cause mortality)

The incidence of each primary AE (i.e., AMI, stroke, CV-related mortality, MACE [AMI+stroke+CV-related mortality], VTE, ATE, cancer [excluding non-melanoma skin cancer (NMSC)], NMSC, and all-cause mortality) was analyzed separately. Each AE was defined when the ICD-10 code was coded at least twice as the first or second diagnosis code. CV-related mortality was confirmed if the patient had a main diagnosis ICD-10 code starting with “I” immediately before death occurred. The ICD-10 codes for all the outcomes are listed in Supplementary Material 1. The incidence rates (IRs) of AEs 1 year after JAKi or TNFi initiation and after the full follow-up period was completed were calculated separately.

### Statistical analysis

Categorical variables were compared using the chi-square test, and the frequency and percentage data were presented. Continuous variables were presented as mean± standard deviation (SD). IRs were expressed as events per 100 person-years (PYs). The TNFi group was selected as the reference group for calculating the incidence rates ratio (IRR) of primary outcomes in the JAKi group. Adjusted HRs for each primary outcome in sets 1 and 2 were measured using multivariate Cox regression analysis, adjusting for various factors such as medication use, age, sex, and presence of comorbidities. Time-dependent covariate tests revealed no violations of the proportional hazards assumption. A two-tailed p-value <0.05 was considered to indicate statistical significance. All tests were performed using SAS Enterprise software, version 7.3 (SAS Institute, Inc., Cary, NC, USA).

### Ethics statement

This study was conducted in accordance with the Declaration of Helsinki and its amendments. The requirement for written informed consent was waived by the Institutional Review Board of Konkuk University Medical Center due to the characteristics of the NHIS data. This study was approved by the Institutional Review Board of Konkuk University Medical Center (approval No. KUMC 2022-02-024).

## RESULTS

### Baseline demographics of Janus kinase inhibitor and tumor necrosis factor inhibitor groups

A total of 1,596 patients with RA (645 in the JAKi group and 951 in the TNFi group) were included in set 1, and 11,765 patients with RA (2,498 in the JAKi group and 9,267 in the TNFi group) were included in set 2. The mean total follow-up duration was 2.1 years for the JAKi group and 2.5 years for the TNFi group in set 1, and 2.6 years for the JAKi group and 5.8 years for the TNFi group in set 2. The baseline characteristics of sets 1 and 2 are summarized in Tables 1 and 2, respectively.

### Incidence rates and incidence rate ratios of acute myocardial infarction, stroke, cardiovascular-related mortality, major adverse cardiovascular event, all-cause mortality, venous thromboembolism, arterial thromboembolism, and cancer in patients with rheumatoid arthritis receiving Janus kinase inhibitors or tumor necrosis factor inhibitors

The IRs per 100 PYs for each outcome in sets 1 and 2 are summarized in Supplementary Material 4 and 5, respectively. IRRs were calculated by comparing the IR of each AE in the JAKi group to the IR of the corresponding outcome in the TNFi group (the reference group). In set 1, the IRRs 1 year after initiation of JAKi for AMI, stroke, CV-related mortality, MACE, all-cause mortality, and cancers other than NMSC were 0.95 (95% confidence interval [CI], 0.41 to 2.19), 0.27 (95% CI, 0.06 to 1.20), 1.47 (95% CI, 0.43 to 5.09), 0.71 (95% CI, 0.37 to 1.34), 1.23 (95% CI, 0.38 to 4.03), and 0.80 (95% CI, 0.30 to 2.17), respectively. The IRRs for the total follow-up duration in the JAKi group were also non-significant for all the events in set 1 (Table 3). In set 2, IRRs for the 1-year follow-up were 0.62 (95% CI, 0.41 to 0.93) for AMI, 0.55 (95% CI, 0.31 to 0.96) for stroke, 0.74 (95% CI, 0.56 to 0.98) for MACE, and 0.57 (95% CI, 0.38 to 0.87) for cancers other than NMSC; the IRRs for other events were non-significant (Table 4). The IRRs for AMI, MACE, and cancers other than NMSC after the 1-year follow-up were significantly lower in women, whereas the IRR for VTE was significantly higher (IRR, 1.83; 95% CI, 1.00 to 3.33). For the full follow-up duration of set 2, the IRRs for AMI in all patients with RA (IRR, 0.69; 95% CI, 0.53 to 0.90) and in women, in particular (IRR, 0.73; 95% CI, 0.55 to 0.98), were significantly lower in the JAKi group; whereas the IRR for VTE was significantly higher in women (IRR, 1.49; 95% CI, 1.00 to 2.21) within this treatment group (Table 4).

### Comparison of hazards for acute myocardial infarction, stroke, cardiovascular-related mortality, major adverse cardiovascular event, all-cause mortality, venous thromboembolism, arterial thromboembolism, and cancer between the Janus kinase inhibitor and tumor necrosis factor inhibitor groups

The HRs for each AE were calculated using multivariate Cox regression analysis. In set 1, the HRs were 0.79 (95% CI, 0.33 to 1.87) for AMI, 0.31 (95% CI, 0.07 to 1.43) for stroke, 0.21 (95% CI, 0.04 to 1.21) for CV-related mortality, 0.64 (95% CI, 0.33 to 1.22) for MACE, 0.97 (95% CI, 0.29 to 3.28), for all-cause mortality, and 0.68 (95% CI, 0.25 to 1.88) for cancers other than NMSC 1-year after JAKi or TNFi initiation (Table 5).

For the full duration of follow-up, the HRs were 0.65 (95% CI, 0.34 to 1.27) for AMI, 0.43 (95% CI, 0.14 to 1.33) for stroke, 1.26 (95% CI, 0.47 to 3.40) for CV-related mortality, 1.41 (95% CI, 0.65 to 3.04) for all-cause mortality, 0.33 (95% CI, 0.07 to 1.54) for VTE, 1.58 (95% CI, 0.10 to 25.47) for ATE, and 1.29 (95% CI, 0.68 to 2.45) for cancers other than NMSC. The total follow-up HRs for MACE were significantly lower in the JAKi group of set 1 (HR, 0.59; 95% CI. 0.35 to 0.99) (Table 5).

The HRs for each event after the 1-year and total follow-up periods for set 2 are summarized in Table 6. After the 1-year follow-up period, HRs for AMI (HR, 0.65; 95% CI, 0.43 to 0.99) and cancers other than NMSC (HR, 0.59; 95% CI, 0.39 to 0.89) were significantly lower in the JAKi group than in the TNFi group, whereas the HR for CV-related mortality was higher in the JAKi group (HR, 1.66; 95% CI, 1.05 to 2.63). The HRs for the other events were not significant. In the age-specific subgroup analysis of patients aged 65 years or over, the JAKi treatment was associated with a significantly higher HR for CV-related mortality (HR, 1.85; 95% CI, 1.05 to 3.28), whereas the HRs for other events were not significant. The HRs for CV-related mortality were higher in the JAKi group than in the TNFi group; however, the HRs for MACE, which included AMI, stroke, and CV-related mortality, were non-significant. For the full duration of follow-up of set 2 patients, HRs for AMI and MACE were significantly lower in the JAKi group than in the TNFi group. The AMI-specific HR in the subgroup of patients aged 65 years or over was also significantly lower in the JAKi group. The HRs for all-cause mortality were significantly higher in the JAKi group than in the TNFi group, for all patients with RA (HR, 1.71; 95% CI, 1.32 to 2.22) and in the subgroup of patients aged 65 years or over (HR, 1.88; 95% CI, 1.36 to 2.60).

## DISCUSSION

The ORAL Surveillance study had a considerable impact on the mode of RA treatment [12]. The use of JAKis has only been permitted in patients who were refractory to other RA medications. The ORAL Surveillance study was the first to show the potential risks associated with tofacitinib use, particularly with respect to serious AEs such as MACE, cancers, and death. However, the incidence of serious AEs could differ according to the ethnicity of patients and the specificity of each JAKi. Furthermore, real-world data showed conflicting results with ORAL Surveillance [13,14], and risk evaluation of serious AEs raised by ORAL Surveillance was not conducted in Asia, including Korea. The warning of CV-related death in febuxostat is one example of mistaken conclusions based on insufficient evidence. The Cardiovascular Safety of Febuxostat and Allopurinol in Patients with Gout and Cardiovascular Morbidities trial claimed that CV-related mortality was higher with febuxostat than with allopurinol in gout patients [18], but this result was contradicted by the Febuxostat versus Allopurinol Streamlined Trial, which was conducted with a longer follow-up period [19]. Therefore, changing the indication and limiting the use of JAKis in Korea should be reconsidered after more JAKi treatment data are collected.

The present study investigated the IRs of AMI, stroke, CV-related death, MACE, all-cause mortality, VTE, ATE, and cancer in patients with RA receiving JAKis. We found that these IRs were similar to those of RA patients being treated with TNFis. This was true for both bDMARD-naive or JAKi-naive newly diagnosed RA patients (set 1) and all RA patients (set 2). Moreover, the HRs for each AE in the JAKi user group were not increased for the bDMARD-native or JAKi-naive patients with RA after the 1-year follow-up period. In fact, the total follow-up HR for MACE was significantly lower in the JAKi treatment group. The HRs for MACE and the components of MACE (i.e., AMI and stroke) were significantly lower in the JAKi group than in the TNFi group; whereas the HR for all-cause mortality was significantly increased in the JAKi group of set 2. The present study used real-world data to compare CV-related outcomes, mortality, thromboembolism, and cancer occurrence between RA patients treated with JAKis versus TNFis. Furthermore, we calculated IRs, IRRs, and HRs for 2 follow-up periods (1 year and total), because some of the AEs (e.g., AMI, stroke, CV-related mortality, MACE, VTE, and ATE) can occur soon after the initiation of treatment.

Patients with RA have an increased risk of CVD and CV-related mortality [2,20,21]. Although several bDMARDs appear to have little effect on CVD prevention [22-27], most bDMARDs (including TNFis) are associated with a reduced risk of CVD compared with csDMARDs [28-31]. A meta-analysis of 26 randomized clinical trials of various JAKis demonstrated no significant difference regarding the occurrence of CVD or MACE compared with the placebo or csDMARDs [32]. The ORAL Surveillance study compared MACE risk between the tofacitinib (5 mg twice daily or 10 mg twice daily) group and the TNFi group and found that tofacitinib use significantly increased the HR for MACE [12]. However, this study included patients with RA who were aged 50 years and over with at least one CVD risk factor; moreover, only 4% of the enrolled patients were Asian [12]. The post hoc analysis of ORAL Surveillance demonstrated that the risk of MACE was slightly increased in the tofacitinib group of RA patients with a history of ASCVD, but not in RA patients without ASCVD [15]. In addition, analyses of real-world data from the United States and France showed non-significance between JAKi and TNFi with respect to MACE risk (HR, 1.01; 95% CI 0.83 to 1.23 and HR, 1.0; 95% CI, 0.7 to 1.6, respectively) [13,14]. The mortality rates associated with heart disease differ according to ethnicity, and are considerably lower in Asian or Pacific Islander populations than in other ethnic groups (e.g., Black, Caucasian, or Hispanic) [33]. In the present study, the risk of CVD (including AMI, stroke, CV-related mortality, and MACE) was not increased in the JAKi group. These results contradict findings from the ORAL Surveillance study [12]. Further studies should be conducted to clarify the potential of JAKi for inducing CV-related events.

Patients with RA have an increased risk of VTE [5,34]. A study of data from the Truven MarketScan and US Medicare claims databases demonstrated no significant differences between tofacitinib and TNFi use regarding VTE risk (HR, 1.33; 95% CI, 0.78 to 2.24) [6]. In addition, a meta-analysis of 42 phase II and III randomized controlled trials of JAKi use for various inflammatory diseases (including RA, ankylosing spondylitis, psoriatic arthritis, psoriasis, Crohn’s disease, and ulcerative colitis) revealed that the JAKi-associated IRR for VTE was 0.68 (95% CI, 0.36 to 1.29) [35]. A recent analysis of French health data also observed no increased risk of VTE in the JAKi group (tofacitinib and baricitinib) versus the TNFi (adalimumab) group (HR, 1.1; 95% CI, 0.7 to 1.6) [14]. In the present study, the IRRs for VTE in set 2 were significantly higher in women than in men (Table 4), and the HR indicated an increased risk in a specific subgroup of the JAKi group (women in set 2, Table 6). The overall HRs for VTE showed no significant differences between the JAKi and TNFi groups in sets 1 and 2. In addition, the ORAL Surveillance study showed that the HRs for VTE only demonstrated significant increased risk at the higher dose of tofacitinib compared with the TNFi group (HR, 3.52; 95% CI, 1.74 to 7.12) [12]. The dose-dependency of risk was not evaluated in the present study, because only the lower dose of tofacitinib (5 mg twice daily) is approved for use in Korea.

A meta-analysis of 13 randomized control trials comparing JAKi+MTX and MTX monotherapy revealed that the relative risks for cancer and NMSC were 1.42 (95% CI, 0.59 to 3.41) and 1.44 (95% CI, 0.36 to 5.76), respectively, in the JAKi+MTX group [36]. The ORAL Surveillance study resulted in an HR for cancer (excluding NMSC) of 1.48 (95% CI, 1.04 to 2.09) in the combined tofacitinib group (5 mg twice daily and 10 mg twice daily) and HRs for NMSC of 1.90 (95% CI, 1.04 to 3.47) and 2.16 (95% CI, 1.19 to 3.92) in the tofacitinib 5 mg and 10 mg twice daily groups, respectively, when compared with the TNFi group [12]. The incidence and mortality rates for cancer vary by ethnicity [37,38]. In the present study, the HR for cancer was not significantly higher in the JAKi group than in the TNFi group.

The present study had several limitations. First, insurance claims do not record important clinical information about patients with RA, such as the presence of rheumatoid factor/anti-citrullinated antibody, erythrocyte sedimentation rate, C-reactive protein, disease activity parameters (such as DAS28), disease duration, and history of bDMARD use. Second, the important factors for predicting malignancy, CVD, and mortality, such as smoking, alcohol, physical activity, obesity, and socioeconomic status, were not included in the present analysis. In addition, some factors known to influence CVD, such as age, glucocorticoid/NSAID use, and seropositivity, varied between the JAKi and TNFi groups (Tables 1 and 2). Although multivariate regression analysis was performed after adjusting for these factors, these differences in baseline characteristics are intrinsic limitations of this observational study. Further research designed as a randomized, prospective study similar to the ORAL Surveillance study should be conducted to overcome these limitations and reconfirm the results of the present study. Third, the JAKi group included considerably fewer RA patients than the TNFi group because the first JAKi was only approved for use in RA patients since March 2015 as a second-line treatment for TNFi non-responders. Meanwhile, tofacitinib was only approved as a first-line therapy for csDMARD non-responders in July 2017. Furthermore, we did not subdivide JAKi users according to each specific JAKi due to the relatively small size of the JAKi group. We analyzed bDMARD-native and JAKi-naive newly diagnosed RA patients (set 1) alongside all RA patients who were treated with JAKi or TNFi (set 2) to overcome the relatively small number of bDMARD-native and JAKi-naive RA patients (set 1). Fourth, the follow-up duration was too short to reveal the risk of malignancy and CVD. The duration between exposure to a carcinogen to the development of malignancy is assumed to be at least 10 years, and risk estimators for CVD usually evaluate 10-year risks for CVD. Fifth, the relatively low incidences of AMI, stroke, CV-related mortality, and ATE could affect the associated HRs of JAKi use. The data of JAKi-related serious AEs have been gathered since 2015, and further studies, including longer duration, may overcome the fourth and fifth limitations of the present study.

In conclusion, the overall risks of AMI, stroke, CV-related mortality, MACE, VTE, ATE, and cancer were similar between the JAKi and TNFi treatment groups of Korean patients with RA. On the other hand, the risk of all-cause mortality was increased in RA patients with JAKi versus TNFi treatment. Further studies are necessary to clarify this discrepancy between all-cause mortality and other serious AEs among RA patients with JAKi. Furthermore, the currently imposed restriction of JAKi use should be supported by further country-specific evidence confirming its association with MACE, thromboembolism, cancer, and CV-related mortality in patients with RA.

## Figures and Tables

**Figure f1-epih-45-e2023045:**
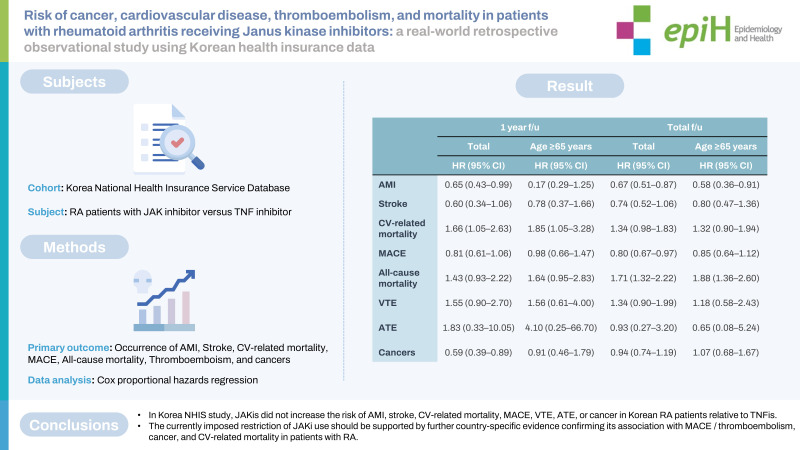


**Table 1.** Baseline characteristics of patients with RA receiving JAKis or TNFis (set 1)

Characteristics	RA with JAKis (n=645)	RA with TNFis (n=951)	p-value
Women	487 (75.5)	688 (72.3)	0.16
Seropositive RA	537 (83.3)	727 (76.4)	<0.01
Age, mean±SD (yr)	52.30±12.80	50.16±14.44	<0.01
Age (yr)			<0.01
18-29	34 (5.3)	103 (10.8)	
30-39	71 (11.0)	130 (13.7)	
40-49	147 (22.8)	181 (19.0)	
50-59	206 (31.9)	284 (29.9)	
60-69	130 (20.1)	170 (17.9)	
≥70	57 (8.8)	83 (8.7)	
Medication			
Tofacitinib	279 (43.3)	-	
Baricitinib	364 (56.4)	-	
Upadacitinib	2 (0.3)	-	
Adalimumab	-	319 (33.5)	
Infliximab	-	107 (11.2)	
Etanercept	-	253 (26.6)	
Golimumab	-	272 (28.6)	
Glucocorticoid	602 (93.3)	815 (85.7)	<0.01
NSAID	605 (93.8)	891 (93.7)	0.93
Comorbidity			
HTN	155 (24.0)	232 (24.40)	0.87
DM	111 (17.2)	149 (15.7)	0.41
CKD	7 (1.1)	6 (0.6)	0.32
Heart failure	9 (1.4)	8 (0.8)	0.29

Values are presented as number (%). RA, rheumatoid arthritis; JAKi, Janus kinase inhibitor; TNFi, tumor necrosis factor inhibitor; SD, standard deviation; NSAID, non-steroidal anti-inflammatory drug; HTN, hypertension; DM, diabetes mellitus; CKD, chronic kidney disease.

**Table 2.** Baseline characteristics of patients with RA receiving JAKis or TNFis (set 2)

Characteristics	RA with JAKis (n=2,498)	RA with TNFis (n=9,267)	p-value
Women	2,078 (83.2)	7,591 (81.9)	0.14
Seropositive RA	2,193 (87.8)	8,190 (88.4)	0.42
Age, mean±SD	51.50±12.25	52.00±13.27	0.07
Age (yr)			<0.01
18-29	113 (4.5)	547 (5.9)	
30-39	307 (12.3)	1,158 (12.5)	
40-49	607 (24.3)	1,950 (21.0)	
50-59	807 (32.3)	2,926 (31.6)	
60-69	496 (19.8)	1,850 (20.0)	
≥70	168 (6.7)	836 (9.0)	
Medication			
Tofacitinib	1,431 (57.3)	-	
Baricitinib	1,061 (42.5)	-	
Upadacitinib	6 (0.2)	-	
Adalimumab	-	3,337 (36.0)	
Infliximab	-	1,381 (14.9)	
Etanercept	-	3,034 (32.7)	
Golimumab	-	1,515 (16.3)	
Glucocorticoid	2,259 (90.4)	7,962 (85.9)	<0.01
NSAID	2,309 (92.4)	8,383 (90.5)	<0.01
Comorbidity			
HTN	678 (27.1)	2,559 (27.6)	0.64
DM	398 (15.9)	1,337 (14.4)	0.06
CKD	27 (1.1)	72 (0.8)	0.14
Heart failure	38 (1.5)	109 (1.2)	0.17

Values are presented as number (%). RA, rheumatoid arthritis; JAKi, Janus kinase inhibitor; TNFi, tumor necrosis factor inhibitor; SD, standard deviation; NSAID, non-steroidal anti-inflammatory drug; HTN, hypertension; DM, diabetes mellitus; CKD, chronic kidney disease.

**Table 3.** Incidence rate ratios for AMI, stroke, CV-related mortality, MACE, all-cause mortality, VTE, ATE, and cancer in patients with RA receiving JAKis (compared with TNFi users) in set 1

Variables	Overall		Men		Women	
	1-yr follow-up	Total follow-up	1-yr follow-up	Total follow-up	1-yr follow-up	Total follow-up
AMI	0.95 (0.41, 2.19)	0.77 (0.40, 1.48)	0.55 (0.06, 5.30)	0.26 (0.03, 2.09)	1.03 (0.41, 2.55)	0.92 (0.45, 1.86)
Stroke	0.27 (0.06, 1.20)	0.44 (0.15, 1.33)	NA	0.84 (0.16, 4.34)	0.40 (0.08, 1.94)	0.30 (0.07, 1.37)
CV-related mortality	1.47 (0.43, 5.09)	1.43 (0.57, 3.63)	NA	1.41 (0.24, 8.42)	1.77 (0.47, 6.58)	1.45 (0.49, 4.32)
MACE	0.71 (0.37, 1.34)	0.69 (0.42, 1.14)	0.20 (0.03, 1.63)	0.55 (0.18, 1.66)	0.87 (0.44, 1.74)	0.73 (0.41, 1.29)
All-cause mortality	1.23 (0.38, 4.03)	1.46 (0.70, 3.03)	1.67 (0.23, 11.83)	1.42 (0.40, 5.02)	1.06 (0.24, 4.73)	1.52 (0.62, 3.74)
VTE	NA	0.36 (0.08, 1.62)	NA	NA	NA	0.42 (0.09, 1.97)
ATE	NA	1.79 (0.11, 28.62)	NA	NA	NA	1.69 (0.11, 27.00)
Cancer (excluding non-melanoma skin cancer)	0.80 (0.30, 2.17)	1.53 (0.81, 2.87)	0.33 (0.04, 2.84)	0.83 (0.26, 2.66)	1.18 (0.36, 3.86)	2.15 (0.98, 4.73)
Non-melanoma skin cancer	NA	NA	NA	NA	NA	NA

Values are presented as incidence rate ratio (95% confidence interval). AMI, acute myocardial infarction; CV, cardiovascular; MACE, major adverse cardiovascular event; VTE, venous thromboembolism; ATE, arterial thromboembolism; RA, rheumatoid arthritis; JAKi, Janus kinase inhibitor; TNFi, tumor necrosis factor inhibitor; NA, not applicable.

**Table 4.** Incidence rate ratios for AMI, stroke, CV-related mortality, MACE, all-cause mortality, VTE, ATE, and cancer in patients with RA receiving JAKis (compared with TNFi users) in set 2

Variables	Overall		Men		Women	
	1-yr follow-up	Total follow-up	1-yr follow-up	Total follow-up	1-yr follow-up	Total follow-up
AMI	0.62 (0.41, 0.93)	0.69 (0.53, 0.90)	0.73 (0.33, 1.63)	0.59 (0.34, 1.03)	0.59 (0.36, 0.95)	0.73 (0.55, 0.98)
Stroke	0.55 (0.31, 0.96)	0.74 (0.52, 1.05)	0.57 (0.20, 1.62)	0.80 (0.39, 1.65)	0.54 (0.28, 1.06)	0.73 (0.49, 1.08)
CV-related mortality	1.44 (0.92, 2.27)	1.14 (0.85, 1.54)	1.61 (0.77, 3.35)	1.18 (0.68, 2.06)	1.39 (0.78, 2.48)	1.15 (0.81, 1.64)
MACE	0.74 (0.56, 0.98)	0.84 (0.70, 1.00)	0.85 (0.52, 1.39)	0.84 (0.58, 1.21)	0.71 (0.51, 0.99)	0.84 (0.69, 1.04)
All-cause mortality	1.32 (0.86, 2.03)	1.19 (0.94, 1.52)	1.29 (0.63, 2.63)	1.08 (0.68, 1.71)	1.37 (0.80, 2.36)	1.27 (0.95, 1.69)
VTE	1.52 (0.88, 2.63)	1.35 (0.92, 1.97)	0.66 (0.15, 2.97)	0.56 (0.13, 2.34)	1.83 (1.00, 3.33)	1.49 (1.00, 2.21)
ATE	1.86 (0.34, 10.13)	0.87 (0.27, 2.86)	NA	NA	1.83 (0.33, 9.98)	1.24 (0.37, 4.19)
Cancer (excluding non-melanoma skin cancer)	0.57 (0.38, 0.87)	1.02 (0.81, 1.29)	0.51 (0.20, 1.29)	0.72 (0.41, 1.26)	0.60 (0.38, 0.95)	1.12 (0.87, 1.45)
Non-melanoma skin cancer	NA	1.10 (0.33, 3.66)	NA	2.27 (0.25, 20.28)	NA	0.87 (0.20, 3.75)

Values are presented as incidence rate ratio (95% confidence interval). AMI, acute myocardial infarction; CV, cardiovascular; MACE, major adverse cardiovascular event; VTE, venous thromboembolism; ATE, arterial thromboembolism; RA, rheumatoid arthritis; JAKi, Janus kinase inhibitor; TNFi, tumor necrosis factor inhibitor; NA, not applicable.

**Table 5.** Multivariate Cox proportional regression analysis of JAKi (compared to TNFi) use on the risk of AMI, stroke, CV-related mortality, MACE, all-cause mortality, VTE, ATE, and cancer occurrence during the 1-year and total follow-up periods (set 1)^1^

Variables		1-yr follow-up				Total follow-up			
		Total	p-value	Age ≥65 yr	p-value	Total	p-value	Age ≥65 yr	p-value
AMI									
	Overall	0.79 (0.33, 1.87)	0.59	1.44 (0.25, 8.26)	0.68	0.65 (0.34, 1.27)	0.21	1.21 (0.39, 3.75)	0.74
	Men	0.59 (0.06, 5.77)	0.65	NA		0.27 (0.03, 2.20)	0.22	NA	
	Women	0.79 (0.30, 2.05)	0.63	1.44 (0.25, 8.26)	0.68	0.69 (0.33, 1.45)	0.33	1.48 (0.44, 4.99)	0.53
Stroke									
	Overall	0.31 (0.07, 1.43)	0.13	0.28 (0.03, 2.44)	0.25	0.43 (0.14, 1.33)	0.14	0.18 (0.02, 1.49)	0.11
	Men	NA		NA		0.76 (0.13, 4.34)	0.76	NA	
	Women	0.46 (0.09, 2.31)	0.35	0.40 (0.04, 3.82)	0.43	0.30 (0.07, 1.39)	0.12	0.22 (0.03, 1.89)	0.17
CV-related mortality									
	Overall	0.21 (0.04, 1.21)	0.08	0.60 (0.13, 2.84)	0.52	1.26 (0.47, 3.40)	0.64	1.10 (0.33, 3.62)	0.88
	Men	NA		NA		1.35 (0.21, 8.65)	0.75	0.77 (0.04, 15.93)	0.87
	Women	1.06 (0.25, 4.40)	0.94	0.66 (0.13, 3.26)	0.61	1.12 (0.34, 3.68)	0.86	0.87 (0.24, 3.12)	0.83
MACE									
	Overall	0.64 (0.33, 1.22)	0.18	0.66 (0.25, 1.74)	0.40	0.59 (0.35, 0.99)	0.04	0.66 (0.31, 1.40)	0.28
	Men	0.23 (0.03, 1.84)	0.16	NA		0.53 (0.17, 1.63)	0.27	0.52 (0.05, 5.01)	0.57
	Women	0.72 (0.35, 1.48)	0.38	0.79 (0.28, 2.22)	0.66	0.57 (0.31, 1.03)	0.06	0.66 (0.29, 1.51)	0.33
All-cause mortality									
	Overall	0.97 (0.29, 3.28)	0.97	1.65 (0.11, 25.26)	0.72	1.41 (0.65, 3.04)	0.39	1.70 (0.57, 5.06)	0.34
	Men	1.48 (0.21, 10.56)	0.70	NA		1.61 (0.43, 6.01)	0.48	4.16 (0.31, 55.25)	0.28
	Women	0.69 (0.13, 3.56)	0.65	NA		1.20 (0.45, 3.20)	0.71	1.14 (0.34, 3.85)	0.83
VTE									
	Overall	NA		NA		0.33 (0.07, 1.54)	0.16	NA	
	Men	NA		NA		NA		NA	
	Women	NA		NA		0.42 (0.09, 2.03)	0.28	NA	
ATE									
	Overall	NA		NA		1.58 (0.10, 25.47)	0.75	NA	
	Men	NA		NA		NA		NA	
	Women	NA		NA		1.58 (0.10, 25.47)	0.75	NA	
Cancer (excluding non-melanoma skin cancer)									
	Overall	0.68 (0.25, 1.88)	0.46	1.49 (0.37, 6.07)	0.57	1.29 (0.68, 2.45)	0.44	1.65 (0.56, 4.88)	0.37
	Men	0.28 (0.03, 2.48)	0.25	0.74 (0.07, 8.41)	0.81	0.63 (0.19, 2.08)	0.45	0.68 (0.11, 4.11)	0.68
	Women	1.08 (0.33, 3.54)	0.90	1.98 (0.33, 11.96)	0.46	2.00 (0.90, 4.44)	0.09	3.50 (0.67, 18.17)	0.14
Non-melanoma skin cancer									
	Overall	NA		NA		NA		NA	
	Men	NA		NA		NA		NA	
	Women	NA		NA		NA		NA	

Values are presented as hazard ratio (95% confidence interval). JAKi, Janus kinase inhibitor; TNFi, tumor necrosis factor inhibitor; AMI, acute myocardial infarction; CV, cardiovascular; MACE, major adverse cardiovascular event; VTE, venous thromboembolism; ATE, arterial thromboembolism; NA, not applicable.

1 The multivariate analysis of the overall patient study group was adjusted for baseline age, gender, hypertension, diabetes, chronic kidney disease, heart failure, and medications (e.g., glucocorticoids and non-steroidal anti-inflammatory drugs); The multivariate analysis of men and women was adjusted for baseline age, hypertension, diabetes, chronic kidney disease, heart failure, and medications (e.g., glucocorticoids and non-steroidal anti-inflammatory drugs).

**Table 6.** Multivariate Cox proportional regression analysis of JAKi (compared to TNFi) use on the risk of AMI, stroke, CV-related mortality, MACE, all-cause mortality, VTE, ATE, and cancer occurrence during the 1-year and total follow-up periods (set 2)^1^

Variables		1-yr follow-up				Total follow-up			
		Total	p-value	Age ≥65 yr	p-value	Total	p-value	Age ≥65 yr	p-value
AMI									
	Overall	0.65 (0.43, 0.99)	0.04	0.17 (0.29, 1.25)	0.17	0.67 (0.51, 0.87)	<0.01	0.58 (0.36, 0.91)	0.02
	Men	0.75 (0.34, 1.69)	0.49	0.88 (0.25, 3.10)	0.84	0.55 (0.31, 0.98)	0.04	0.33 (0.10, 1.08)	0.07
	Women	0.61 (0.38, 0.99)	0.04	0.49 (0.19, 1.24)	0.13	0.71 (0.53, 0.96)	0.03	0.67 (0.40, 1.10)	0.11
Stroke									
	Overall	0.60 (0.34, 1.06)	0.08	0.78 (0.37, 1.66)	0.52	0.74 (0.52, 1.06)	0.10	0.80 (0.47, 1.36)	0.41
	Men	0.60 (0.21, 1.73)	0.35	0.80 (0.18, 3.67)	0.78	0.71 (0.34, 1.50)	0.37	0.59 (0.18, 1.96)	0.39
	Women	0.61 (0.31, 1.18)	0.14	0.78 (0.33, 1.86)	0.58	0.77 (0.51, 1.15)	0.20	0.90 (0.50, 1.61)	0.71
CV-related mortality									
	Overall	1.66 (1.05, 2.63)	0.03	1.85 (1.05, 3.28)	0.03	1.34 (0.98, 1.83)	0.07	1.32 (0.90, 1.94)	0.15
	Men	2.00 (0.94, 4.26)	0.07	2.66 (1.03, 6.89)	0.04	1.24 (0.69, 2.22)	0.47	1.47 (0.73, 2.95)	0.28
	Women	1.53 (0.86, 2.74)	0.15	1.59 (0.77, 3.29)	0.21	1.38 (0.95, 2.00)	0.09	1.28 (0.80, 2.03)	0.30
MACE									
	Overall	0.81 (0.61, 1.06)	0.13	0.98 (0.66, 1.47)	0.93	0.80 (0.67, 0.97)	0.02	0.85 (0.64, 1.12)	0.24
	Men	0.91 (0.55, 1.51)	0.72	1.25 (0.61, 2.53)	0.54	0.74 (0.51, 1.09)	0.13	0.74 (0.41, 1.07)	0.30
	Women	0.76 (0.54, 1.07)	0.11	0.90 (0.55, 1.47)	0.66	0.83 (0.67, 1.02)	0.08	0.89 (0.65, 1.21)	0.46
All-cause mortality									
	Overall	1.43 (0.93, 2.22)	0.10	1.64 (0.95, 2.83)	0.08	1.71 (1.32, 2.22)	<0.01	1.88 (1.36, 2.60)	<0.01
	Men	1.33 (0.61, 2.75)	0.45	1.53 (0.59, 3.97)	0.39	1.29 (0.79, 2.10)	0.30	1.49 (0.79, 2.81)	0.22
	Women	1.48 (0.85, 2.55)	0.16	1.76 (0.90, 3.46)	0.10	1.92 (1.41, 2.62)	<0.01	2.07 (1.42, 3.03)	<0.01
VTE									0.64
	Overall	1.55 (0.90, 2.70)	0.12	1.56 (0.61, 4.00)	0.35	1.34 (0.90, 1.99)	0.15	1.18 (0.58, 2.43)	
	Men	0.68 (0.15, 3.06)	0.62	0.70 (0.08, 5.92)	0.74	0.46 (0.11, 1.96)	0.29	0.71 (0.09, 5.76)	0.75
	Women	1.86 (1.02, 3.41)	0.04	2.04 (0.69, 5.99)	0.20	1.55 (1.02, 2.36)	0.04	1.28 (0.59, 2.75)	0.53
ATE									
	Overall	1.83 (0.33, 10.05)	0.49	4.10 (0.25, 66.70)	0.32	0.93 (0.27, 3.20)	0.91	0.65 (0.08, 5.24)	0.69
	Men	NA		NA		NA		NA	
	Women	1.83 (0.33, 10.05)	0.49	4.10 (0.25, 66.70)	0.32	1.22 (0.34, 4.38)	0.76	1.02 (0.12, 8.86)	0.99
Cancer (excluding non-melanoma skin cancer)									
	Overall	0.59 (0.39, 0.89)	0.01	0.91 (0.46, 1.79)	0.91	0.94 (0.74, 1.19)	0.61	1.07 (0.68, 1.67)	0.77
	Men	0.50 (0.20, 1.28)	0.15	0.66 (0.19, 2.25)	0.50	0.69 (0.39, 1.24)	0.21	0.44 (0.16, 1.23)	0.12
	Women	0.61 (0.39, 0.97)	0.04	1.03 (0.45, 2.34)	0.95	1.01 (0.78, 1.31)	0.95	1.52 (0.91, 2.54)	0.11
Non-melanoma skin cancer									
	Overall	NA		NA		1.38 (0.39, 4.88)	0.61	1.06 (0.13, 8.80)	0.96
	Men	NA		NA		1.65 (0.17, 16.01)	0.67	3.22 (0.28, 37.41)	0.35
	Women	NA		NA		1.23 (0.27, 5.63)	0.79	NA	

Values are presented as hazard ratio (95% confidence interval). JAKi, Janus kinase inhibitor; TNFi, tumor necrosis factor inhibitor; AMI, acute myocardial infarction; CV, cardiovascular; MACE, major adverse cardiovascular event; VTE, venous thromboembolism; ATE, arterial thromboembolism; NA, not applicable.

1 The multivariate analysis of the overall patient study group was adjusted for baseline age, gender, hypertension, diabetes, chronic kidney disease, heart failure, and medications (e.g., glucocorticoids and non-steroidal anti-inflammatory drugs); The multivariate analysis of men and women was adjusted for baseline age, hypertension, diabetes, chronic kidney disease, heart failure, and medications (e.g., glucocorticoids and non-steroidal anti-inflammatory drugs).

## SUPPLEMENTARY MATERIALS

Supplementary Material 1. ICD-10 codes used to identify patients, outcomes, and baseline comorbidities.

Supplementary Material 2 Flow chart for inclusion and exclusion of enrolled patients in Set 1.

Supplementary Material 3 Flow chart for inclusion and exclusion of enrolled patients in Set 2.

Supplementary Material 4 Incidence rates of AMI, stroke, CV-related mortality, MACE, all-cause mortality, VTE, ATE, and cancer in patients with RA receiving JAKis or TNFis (Set 1).

Supplementary Material 5 Incidence rates of AMI, stroke, CV-related mortality, MACE, all-cause mortality, VTE, ATE, and cancer in patients with RA receiving JAKis or TNFis (Set 2).
