# Supplementary material for: Risk of cancer, cardiovascular disease, thromboembolism, and mortality in patients with rheumatoid arthritis receiving Janus kinase inhibitors: a real-world retrospective observational study using Korean health insurance data
Source: Epidemiol Health. 2023 Apr 15;45:e2023045. doi: 10.4178/epih.e2023045 (PMC10396807; doi:10.4178/epih.e2023045)
Supplement: Supplementary Material 1. — ICD-10 codes used to identify patients, outcomes, and baseline comorbidities. [file epih-45-e2023045-Supplementary-1.docx]

**Supplementary Material 1.** ICD-10 codes used to identify patients, outcomes, and baseline comorbidities.

| **National Patient Register**  **(NPR)** | **ICD-10**  1997–present |
| --- | --- |
| **Rheumatic diagnoses** |  |
| Rheumatoid arthritis | M05, M06.0/2/3/8/9 |
| Ankylosing spondylitis | M45 |
| SLE | M32 |
| Psoriasis and psoriatic arthritis | L40.0/1/2/3/4/5/8/9 |
| Behcet’s disease | M352 |
| Ulcerative colitis | K51 |
| Crohn’s disease | K50 |
| **Outcomes** |  |
| AMI | I21 |
| Stroke | I60-I64 |
| CV-related mortality | Death with main ICD-10 “I” code |
| MACE | I21, I60-64, Death events with main ICD-10 “I” code |
| VTE | I80, I81, I82, I260, I269 |
| ATE | I74 |
| Cancer (excluding non-melanoma skin cancer) | C00-43, C45-97 |
| Non-melanoma skin cancer | C44 |
| **Other comorbidities** |  |
| HTN | I10–I13, I15 |
| DM | E11-E14 |
| CKD | N18–N19, Z49, Z904, Z905, Z992 |
| Heart failure | I42, I50 |

ICD-10: International Classification of Diseases 10^th^ Revision; SLE: systemic lupus erythematosus; AMI: acute myocardial infarction; CV: cardiovascular; MACE: major adverse cardiovascular event; VTE: venous thromboembolism; ATE: arterial thromboembolism; HTN: hypertension; DM: diabetes mellitus; CKD: chronic kidney disease.
