# Supplementary figures and images for: Risk of cancer, cardiovascular disease, thromboembolism, and mortality in patients with rheumatoid arthritis receiving Janus kinase inhibitors: a real-world retrospective observational study using Korean health insurance data
Source: Epidemiol Health. 2023 Apr 15;45:e2023045. doi: 10.4178/epih.e2023045 (PMC10396807; doi:10.4178/epih.e2023045)

**Supplementary Material 2. Flow chart for inclusion and exclusion of enrolled patients in Set 1.**

**
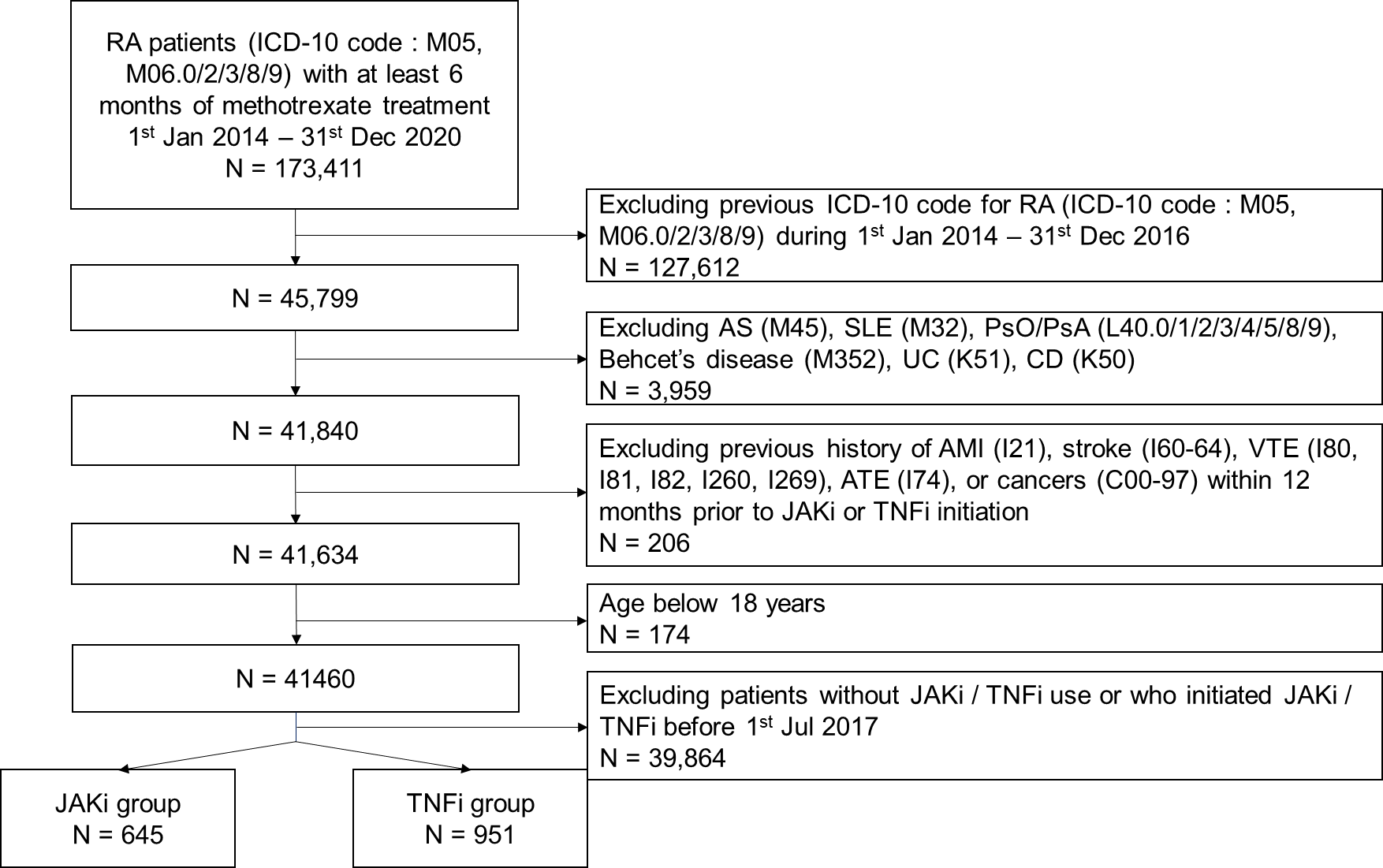
**

Supplement: Supplementary Material 2 — Flow chart for inclusion and exclusion of enrolled patients in Set 1. [file epih-45-e2023045-Supplementary-2.docx]

**Supplementary Material 3. Flow chart for inclusion and exclusion of enrolled patients in Set 2.**


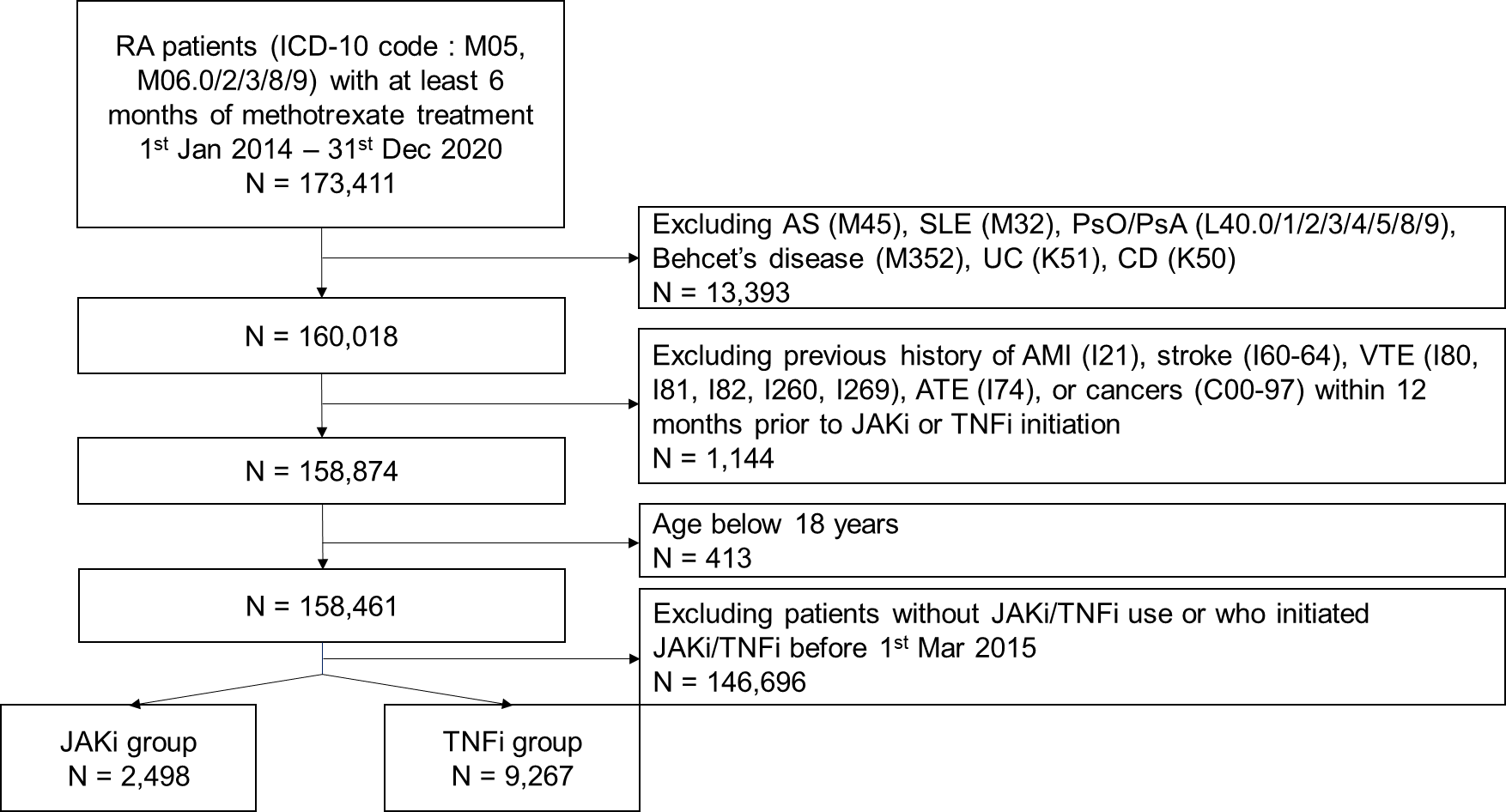

Supplement: Supplementary Material 3 — Flow chart for inclusion and exclusion of enrolled patients in Set 2. [file epih-45-e2023045-Supplementary-3.docx]
