# Supplementary material for: Risk of cancer, cardiovascular disease, thromboembolism, and mortality in patients with rheumatoid arthritis receiving Janus kinase inhibitors: a real-world retrospective observational study using Korean health insurance data
Source: Epidemiol Health. 2023 Apr 15;45:e2023045. doi: 10.4178/epih.e2023045 (PMC10396807; doi:10.4178/epih.e2023045)
Supplement: Supplementary Material 4 — Incidence rates of AMI, stroke, CV-related mortality, MACE, all-cause mortality, VTE, ATE, and cancer in patients with RA receiving JAKis or TNFis (Set 1). [file epih-45-e2023045-Supplementary-4.docx]

**Supplementary Material 4.** Incidence rates of AMI, stroke, CV-related mortality, MACE, all-cause mortality, VTE, ATE, and cancer in patients with RA receiving JAKis or TNFis (Set 1).

|  | RA with JAKis  (n = 645) | | RA with TNFis  (n = 951) | |
| --- | --- | --- | --- | --- |
|  | 1-year follow-up | Total follow-up | 1-year follow-up | Total follow-up |
| **AMI** |  |  |  |  |
| Incident events, n (men/women) | 9 (1/8) | 13 (1/12) | 14 (3/11) | 30 (8/22) |
| PY | 641 | 1358 | 943 | 2419 |
| Overall incidence (n/100 PY) | 1.40 | 0.96 | 1.48 | 1.24 |
| Incidence in men (n/100 PY) | 0.63 | 0.31 | 1.15 | 1.18 |
| Incidence in women (n/100 PY) | 1.66 | 1.16 | 1.61 | 1.26 |
| **Stroke** |  |  |  |  |
| Incident events, n (men/women) | 2 (0/2) | 4 (2/2) | 11 (4/7) | 16 (5/11) |
| PY | 643 | 1370 | 944 | 2433 |
| Overall incidence (n/100 PY) | 0.31 | 0.29 | 1.17 | 0.66 |
| Incidence in men (n/100 PY) | NA | 0.62 | 1.54 | 0.73 |
| Incidence in women (n/100 PY) | 0.41 | 0.19 | 1.02 | 0.63 |
| **CV-related mortality** |  |  |  |  |
| Incident events, n (men/women) | 5 (0/5) | 8 (2/6) | 5 (1/4) | 10 (3/7) |
| PY | 642 | 1361 | 947 | 2439 |
| Overall incidence (n/100 PY) | 0.78 | 0.59 | 0.53 | 0.41 |
| Incidence in men (n/100 PY) | NA | 0.62 | 0.38 | 0.44 |
| Incidence in women (n/100 PY) | 1.03 | 0.58 | 0.58 | 0.40 |
| **MACE** |  |  |  |  |
| Incident events, n (men/women) | 14 (1/13) | 21 (4/17) | 29 (8/21) | 54 (15/39) |
| PY | 638 | 1345 | 935 | 2377 |
| Overall incidence (n/100 PY) | 2.20 | 1.56 | 3.10 | 2.27 |
| Incidence in men (n/100 PY) | 0.63 | 1.24 | 3.10 | 2.25 |
| Incidence in women (n/100 PY) | 2.71 | 1.66 | 3.10 | 2.28 |
| **All-cause mortality** |  |  |  |  |
| Incident events, n (men/women) | 5 (2/3) | 13 (4/9) | 6 (2/4) | 16 (6/10) |
| PY | 642 | 1362 | 947 | 2442 |
| Overall incidence (n/100 PY) | 0.78 | 0.96 | 0.63 | 0.66 |
| Incidence in men (n/100 PY) | 1.27 | 1.24 | 0.76 | 0.88 |
| Incidence in women (n/100 PY) | 0.62 | 0.87 | 0.58 | 0.57 |
| **VTE** |  |  |  |  |
| Incident events, n (men/women) | 0 | 2 (0/2) | 4 (1/3) | 10 (2/8) |
| PY | 645 | 1374 | 948 | 2443 |
| Overall incidence (n/100 PY) | NA | 0.15 | 0.42 | 0.41 |
| Incidence in men (n/100 PY) | NA | NA | 0.38 | 0.29 |
| Incidence in women (n/100 PY) | NA | 0.19 | 0.44 | 0.46 |
| **ATE** |  |  |  |  |
| Incident events, n (men/women) | 1 (0/1) | 1 (0/1) | 0 | 1 (0/1) |
| PY | 644 | 1373 | 950 | 2457 |
| Overall incidence (n/100 PY) | 0.16 | 0.07 | NA | 0.04 |
| Incidence in men (n/100 PY) | NA | NA | NA | NA |
| Incidence in women (n/100 PY) | 0.21 | 0.10 | NA | 0.06 |
| **Cancer (excluding non-melanoma skin cancer)** |  |  |  |  |
| Incident events, n (men/women) | 6 (1/5) | 18 (4/14) | 11 (5/6) | 21 (10/11) |
| PY | 641 | 1358 | 944 | 2421 |
| Overall incidence (n/100 PY) | 0.94 | 1.33 | 1.17 | 0.87 |
| Incidence in men (n/100 PY) | 0.64 | 1.24 | 1.92 | 1.49 |
| Incidence in women (n/100 PY) | 1.03 | 1.35 | 0.88 | 0.63 |
| **Non-melanoma skin cancer** |  |  |  |  |
| Incident events, n (men/women) | 0 | 0 | 0 | 1 (1/0) |
| PY | 645 | 1375 | 950 | 2456 |
| Overall incidence (n/100 PY) | NA | NA | NA | 0.04 |
| Incidence in men (n/100 PY) | NA | NA | NA | 0.15 |
| Incidence in women (n/100 PY) | NA | NA | NA | NA |

Rates are presented as number of events per 100 PY at risk.

RA: rheumatoid arthritis; JAKi: janus kinase inhibitor; TNFi: tumor necrosis factor inhibitor; AMI: acute myocardial infarction; CV: cardiovascular; MACE: major adverse cardiovascular event; VTE: venous thromboembolism; ATE: arterial thromboembolism; PY: person-years; NA: not applicable.
