# Supplementary material for: Risk of cancer, cardiovascular disease, thromboembolism, and mortality in patients with rheumatoid arthritis receiving Janus kinase inhibitors: a real-world retrospective observational study using Korean health insurance data
Source: Epidemiol Health. 2023 Apr 15;45:e2023045. doi: 10.4178/epih.e2023045 (PMC10396807; doi:10.4178/epih.e2023045)
Supplement: Supplementary Material 5 — Incidence rates of AMI, stroke, CV-related mortality, MACE, all-cause mortality, VTE, ATE, and cancer in patients with RA receiving JAKis or TNFis (Set 2). [file epih-45-e2023045-Supplementary-5.docx]

**Supplementary Material 5**. Incidence rates of AMI, stroke, CV-related mortality, MACE, all-cause mortality, VTE, ATE, and cancer in patients with RA receiving JAKis or TNFis (Set 2).

|  | RA with JAKis  (n = 2498) | | RA with TNFis  (n = 9267) | |
| --- | --- | --- | --- | --- |
|  | 1-year follow-up | Total follow-up | 1-year follow-up | Total follow-up |
| **AMI** |  |  |  |  |
| Incident events, n (men/women) | 26 (7/19) | 62 (13/49) | 156 (38/118) | 729 (189/540) |
| PY | 2486 | 6404 | 9177 | 52164 |
| Overall incidence (n/100 PY) | 1.05 | 0.97 | 1.70 | 1.40 |
| Incidence in men (n/100 PY) | 1.68 | 1.26 | 2.30 | 2.13 |
| Incidence in women (n/100 PY) | 0.92 | 0.91 | 1.57 | 1.25 |
| **Stroke** |  |  |  |  |
| Incident events, n (men/women) | 14 (4/10) | 35 (8/27) | 95 (28/67) | 391 (88/303) |
| PY | 2489 | 6453 | 9208 | 53408 |
| Overall incidence (n/100 PY) | 0.56 | 0.54 | 1.03 | 0.73 |
| Incidence in men (n/100 PY) | 0.96 | 0.76 | 1.69 | 0.95 |
| Incidence in women (n/100 PY) | 0.48 | 0.50 | 0.89 | 0.69 |
| **CV-related mortality** |  |  |  |  |
| Incident events, n (men/women) | 26 (10/16) | 49 (14/35) | 67 (25/42) | 358 (107/251) |
| PY | 2477 | 6398 | 9217 | 53476 |
| Overall incidence (n/100 PY) | 1.05 | 0.77 | 0.73 | 0.67 |
| Incidence in men (n/100 PY) | 2.43 | 1.38 | 1.51 | 1.17 |
| Incidence in women (n/100 PY) | 0.78 | 0.65 | 0.56 | 0.57 |
| **MACE** |  |  |  |  |
| Incident events, n (men/women) | 60 (19/41) | 131 (31/100) | 300 (89/211) | 1248 (310/938) |
| PY | 2460 | 6267 | 9090 | 50107 |
| Overall incidence (n/100 PY) | 2.44 | 2.09 | 3.30 | 2.49 |
| Incidence in men (n/100 PY) | 4.66 | 3.12 | 5.49 | 3.72 |
| Incidence in women (n/100 PY) | 2.00 | 1.90 | 2.83 | 2.25 |
| **All-cause mortality** |  |  |  |  |
| Incident events, n (men/women) | 28 (10/18) | 74 (20/54) | 79 (31/48) | 515 (165/350) |
| PY | 2482 | 6400 | 9217 | 53198 |
| Overall incidence (n/100 PY) | 1.13 | 1.16 | 0.86 | 0.97 |
| Incidence in men (n/100 PY) | 2.41 | 1.96 | 1.87 | 1.82 |
| Incidence in women (n/100 PY) | 0.87 | 1.00 | 0.64 | 0.79 |
| **VTE** |  |  |  |  |
| Incident events, n (men/women) | 18 (2/16) | 31 (2/29) | 44 (12/32) | 193 (32/161) |
| PY | 2489 | 6465 | 9236 | 54141 |
| Overall incidence (n/100 PY) | 0.72 | 0.48 | 0.48 | 0.36 |
| Incidence in men (n/100 PY) | 0.48 | 0.19 | 0.72 | 0.34 |
| Incidence in women (n/100 PY) | 0.77 | 0.54 | 0.42 | 0.36 |
| **ATE** |  |  |  |  |
| Incident events, n (men/women) | 2 (0/2) | 3 (0/3) | 4 (0/4) | 29 (9/20) |
| PY | 2495 | 6501 | 9259 | 54717 |
| Overall incidence (n/100 PY) | 0.08 | 0.05 | 0.04 | 0.05 |
| Incidence in men (n/100 PY) | NA | NA | NA | 0.94 |
| Incidence in women (n/100 PY) | 0.10 | 0.06 | 0.05 | 0.04 |
| **Cancer (excluding non-melanoma skin cancer)** |  |  |  |  |
| Incident events, n (men/women) | 26 (5/21) | 81 (13/68) | 167 (39/128) | 648 (157/491) |
| PY | 2482 | 6386 | 9162 | 52339 |
| Overall incidence (n/100 PY) | 1.05 | 1.27 | 1.82 | 1.24 |
| Incidence in men (n/100 PY) | 1.20 | 1.25 | 2.36 | 1.74 |
| Incidence in women (n/100 PY) | 1.02 | 1.27 | 1.70 | 1.13 |
| **Non-melanoma skin cancer** |  |  |  |  |
| Incident events, n (men/women) | 0 | 3 (1/2) | 3 (1/2) | 23 (4/19) |
| PY | 2496 | 6506 | 9259 | 54741 |
| Overall incidence (n/100 PY) | NA | 0.05 | 0.03 | 0.04 |
| Incidence in men (n/100 PY) | NA | 0.10 | 0.06 | 0.04 |
| Incidence in women (n/100 PY) | NA | 0.04 | 0.03 | 0.04 |

Rates are presented as the number of events per 100 PY at risk.

RA: rheumatoid arthritis; JAKi: janus kinase inhibitor; TNFi: tumor necrosis factor inhibitor; AMI: acute myocardial infarction; CV: cardiovascular; MACE: major adverse cardiovascular event; VTE: venous thromboembolism; ATE: arterial thromboembolism; PY: person-years; NA: non applicable.
